# Utilizing Environmental DNA Metabarcoding to Assess Fish‐Based Food Resources in Key Foraging Areas of the Black Stork

**DOI:** 10.1002/ece3.73526

**Published:** 2026-04-20

**Authors:** Xiangguo Yang, Bo Liu, Jiangyue Wu, Xueyong Yuan, Danyang Li, Sen Liu

**Affiliations:** ^1^ Anyang Prevention and Protection of Forestry Resources Center Anyang China; ^2^ Mineral Resources Exploration Center of Hean Geological Bureau Zhengzhou China; ^3^ College of Life Sciences Henan Normal University Xinxiang China

**Keywords:** Anyang, *Ciconia nigra*, eDNA

## Abstract

The Black Stork ( *Ciconia nigra* ) is a widely distributed yet scarce wading bird, predominantly piscivorous, and is classified as a National First‐Class Key Protected Wildlife Species in China. Effective conservation strategies necessitate the monitoring of its food resources. During the migration period of black storks, this study utilized environmental DNA (eDNA) metabarcoding to assess ichthyological resources within the water bodies of key foraging areas, specifically Zhanghe Canyon National Wetland Park and Qixi River National Wetland Park, Anyang City, Henan Province, China. The findings revealed a diverse range of fish species, including some not previously documented locally, with no significant differences in species composition between the two sites. The results suggest that the primary fish resources in the foraging habitats of black storks are species from the orders Siluriformes, Cypriniformes, and Perciformes. To address the risk of false detections inherent to eDNA technology, we implemented a two‐step filtering method, as detailed and discussed in the main text. Based on these findings, we recommend that local governments integrate dynamic monitoring of the identified fish resources into routine conservation planning for black storks and its habitats, thereby ensuring the stability and sustainability of this essential food resource.

## Graphical abstract

This study employed environmental DNA (eDNA) to assess fish‐based food resources in key foraging areas of the Black Stork ( *Ciconia nigra* )—Zhanghe Canyon and Qixi River National Wetland Parks in Henan, China. Results show that the main prey in these habitats are fish from the orders Siluriformes, Cypriniformes, and Perciformes, with no significant difference in species composition between the two sites. It is recommended that local governments incorporate these fish resources into routine monitoring when formulating conservation policies for Black Stork.

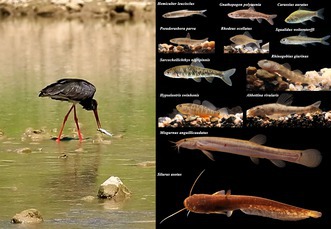

## 1 Introduction

The Black Stork ( *Ciconia nigra* ) is a large wading bird species, classified as “Least Concern” on the IUCN Red List (IUCN 2016); however, its global population remains sparse and fragmented. In China, it's designated as a National First‐Class Key Protected Wildlife Species (NFGA and MARA 2021), highlighting its considerable conservation significance. As a predominantly piscivorous bird, black storks mainly consume fish (Li et al. 2011; Ionescu et al. 2019). Therefore, ensuring the stability and sustainability of fish resources within its foraging habitats is crucial for its conservation.

Traditionally, methods for assessing potential fish food resources, such as netting and electrofishing, have been predominantly employed (Bonar et al. 2009). However, these methods are constrained by several limitations, such as low efficiency, potential harm to organisms, reliance on taxonomic expertise, and an inability to facilitate non‐invasive, high‐frequency monitoring (Yao et al. 2022). In recent years, the advent of environmental DNA (eDNA) metabarcoding technology has introduced innovative opportunities in this domain. eDNA consists of free DNA fragments shed by organisms into environmental matrices like water and soil (Taberlet et al. 2012). This highly sensitive technology addresses numerous challenges in ecology and conservation, including the assessment of species distribution, detection of rare, cryptic, or invasive species, monitoring of ecosystem health and dynamics, and exploration of dietary and trophic interactions (Sahu et al. 2023). By employing eDNA metabarcoding to detect genetic material in aquatic environments, researchers can achieve high‐precision, non‐invasive identification of fish species, often down to the genus or species level (Rees et al. 2014; Garlapati et al. 2019; Yamahara et al. 2025), thereby providing a novel approach for systematically analyzing the foraging ecology of black storks.

Black storks exhibit a preference for foraging in shallow river floodplains adjacent to hilly regions. Anyang City, located in Henan Province, lies within the transitional zone between the eastern foothills of the Taihang Mountains and the plains. This area features extensive floodplains and shallow waters that provide optimal foraging habitats for black storks. Notably, Zhanghe Canyon National Wetland Park in Yindu District and Qixi River National Wetland Park in Linzhou serve as year‐round primary activity regions for black storks, with consistent monthly observations and peak population numbers during the migration period from late October to early November based on our long‐term field observation. Historical records (hereafter referred to as “documented fish”) indicate that Anyang City is home to 53 fish species spanning 43 genera, 19 families, and 7 orders (Zhao et al. 2016; Zhao et al. 2017; Li et al. 2018).

In the end of October 2024, a total of 45 and 18 individuals were found in Qixihe National Wetland Park and Zhanghe National Wetland Park, respectively. During the black stork's aggregation period, this study collected water samples from these core wetlands. Utilizing eDNA metabarcoding technology, a high‐throughput analysis of ichthyological resources was conducted. The objectives of this study are threefold: (1) to compare the results with documented fish and assess the accuracy of eDNA technology for surveying ichthyological resources in Anyang City; (2) to accurately identify the composition and diversity of potential fish food resources in the foraging habitats of black storks, thereby constructing a high‐resolution local fish species inventory; and (3) to provide critical scientific evidence for the precise protection, management, and ecological restoration of black storks' habitat in the Anyang region.

## 2 Materials and Methods

### 2.1 Sample Collection

In the end of October 2024, water sampling was systematically conducted within the primary activity zones of black storks in Qixihe National Wetland Park and Zhanghe National Wetland Park, Anyang City, Henan Province, China. In each park, black storks were mainly concentrated in two activity areas, and we set up three sampling sites in each area, located at the upstream edge, downstream edge, and center point, respectively. To minimize disturbance to the black storks, we collected water samples in the evening after they had departed. During sampling, within a 20 m range of each site, we selected five areas with water depths of approximately 0.3 m and collected 1 L water sample at a depth of approximately 0.15 m below the water surface. These five subsamples were then pooled into one 5 L composite sample representing that site. A total of 12 samples were collected across the two parks. Samples from Qixihe National Wetland Park, collected near the internal tourist attraction WanQuanHu Park, were therefore abbreviated as WQH. Samples from Zhanghe National Wetland Park were abbreviated as ZH. The water samples were filtered through a glass vacuum filtration unit fitted with a 47 mm diameter, 0.45 μm pore size nylon membrane filters (Cat No. 7141‐104, Whatman, Cytiva, USA). Filtration was performed using a vacuum pump. All experimental equipment was sterilized with a 10% bleach solution, achieving an approximate final sodium hypochlorite concentration of 1%, and subsequently rinsed with ddH₂O to prevent contamination by eDNA. Filters containing the retained particulate matter were stored at −20°C for further analysis.

### 2.2 DNA Extraction, PCR Amplification, Library Preparation, and Sequencing

Genomic DNA was extracted from the filters utilizing the MagBeads FastDNA Kit for Soil (MP Biomedicals). The extracted DNA served as the template for the polymerase chain reaction (PCR) amplification of the fish cytochrome c oxidase subunit I (COI) gene, employing the primer pair MlCOIintF (5′‐GGWACWGGWTGAACWGTWTAYCCYCC‐3′) and JgHCO2198 (5′‐TAIACYTCIGGRTGICCRAARAAYCA‐3′) (Leray et al. 2013). Each 20 μL PCR reaction mixture comprised 4 μL of 5× FastPfu Buffer, 2 μL of 2.5 mM dNTPs, 0.8 μL of each primer, 0.4 μL of FastPfu Polymerase, approximately 10 ng of DNA template, and ddH₂O to the final volume. The amplification protocol included an initial denaturation step at 95°C for 5 min, followed by 30 cycles of denaturation at 95°C for 30 s, annealing at 58°C for 30 s, and extension at 72°C for 45 s, with a final extension at 72°C for 10 min. The PCR products were verified via 2% agarose gel electrophoresis, excised, and subsequently purified using the AxyPrep DNA Gel Extraction Kit (Axygen Biosciences, Union City, CA, USA), followed by elution in Tris–HCl buffer. The purified products were quantified using the QuantiFluor ‐ST blue fluorescence quantification system (Promega). Sequencing libraries were constructed and subjected to high‐throughput sequencing using the Illumina PE300 platform. The raw sequencing data have been deposited into the Genome Sequence Archive (Zhang et al. 2025) in the National Genomics Data Center of China (CNCB‐NGDC Members and Partners 2025) (GSA: CRA034477) that are publicly accessible at https://ngdc.cncb.ac.cn/gsa.

### 2.3 Data Processing and Statistical Analysis

The raw sequencing data were processed and subjected to quality control utilizing the DADA2 pipeline within the QIIME2 framework (Callahan et al. 2016). Quality assessment criteria dictated the exclusion of reads if the terminal base quality fell below a score of 20, the average quality score within any 10 bp sliding window was beneath this threshold, or the sequence length was reduced to less than 50 bp post‐quality control. Additionally, sequences containing ambiguous “N” bases were eliminated. Sample identification was achieved through barcodes and primers positioned at the sequence termini, permitting no mismatches for barcodes and allowing up to two mismatches for primers. Paired‐end reads were merged into a single sequence, necessitating a minimum overlap of 10 bp, with reads exhibiting a mismatch rate exceeding 0.2 within the overlapping region being discarded. In contrast to conventional OTU clustering at predefined similarity thresholds (e.g., 97%), DADA2 distinguishes amplicon sequence variants (ASVs) at single‐nucleotide resolution. Identical sequences were consolidated into the same ASV, resulting in the generation of an ASV table and an abundance matrix for subsequent diversity analyses.

Sequences from all samples were rarefied to an even depth to facilitate subsequent analyses. For taxonomic annotation, the ASV sequences were compared against the MitoFish database, the Fish COI gene database (FishCOI), and the NCBI GenBank database. To ensure data reliability, only annotations consistently corroborated across all databases were retained. A conservative filtering strategy was then applied. First, ASVs with a total read count of less than 5 (a threshold of 10^−5^ of the total number of reads) across all samples were excluded to reduce background noise. Next, the remaining ASVs were cross‐referenced with the “Fauna Sinica: Osteichthyes” and local fish surveys (Zhao et al. 2016; Zhao et al. 2017; Li et al. 2018). ASVs identified as species endemic to regions outside the study area were excluded. The retained ASVs, consistently assigned to freshwater fish species known to occur in the study region, were used for subsequent diversity and composition analyses and are hereafter referred to as “ASVs fish”.

Hill numbers were employed to evaluate alpha diversity, as they corporate both relative abundance and species richness, thus circumventing the limitations associated with multiple diversity indices that are frequently highly correlated, expressed in non‐intuitive units, and challenging to compare directly (Chao et al. 2014). There numbers have been effectively utilized in diversity analyses based on DNA sequencing, including studies on microbiomes, dietary analysis, and ecosystem biodiversity assessments (Alberdi and Gilbert 2019). The calculation of Hill numbers was performed using the iNEXT package (Hsieh et al. 2016). To determine significant differences between groups, Kruskal–Wallis rank sum tests were employed.

Beta diversity analysis was conducted using Bray‐Curtis distances with Principal Coordinates Analysis (PCoA) employed to visually depict the dissimilarities in fish community composition among groups. The statistical significance of intergroup differences was evaluated using both Analysis of Similarities (ANOSIM) and Permutational Multivariate Analysis of Variance (PERMANOVA). Furthermore, permutational analysis of multivariate dispersion (PERMDISP) was performed to assess the homogeneity of group variances.

Comparisons were made of the intergroup differences in fish relative abundance at the Order, Family, and Genus levels. For each taxonomic level, the relative abundance of a given taxon in a sample was calculated as the sum of reads assigned to all ASVs belonging to that taxon, divided by the total number of reads in that sample. To identify biomarkers for each group, Linear Discriminant Analysis Effect Size (LEfSe) analysis (Segata et al. 2011) was conducted, employing a Linear Discriminant Analysis (LDA) score threshold greater than 2 and a *p* value of less than 0.05 to determine significant biomarkers based on the Kruskal–Wallis sum‐rank test. All statistical analyses were performed using R (version 4.5.2).

## 3 Results

### 3.1 Comparison With ASVs Fish and Documented Fish

**FIGURE 1:**
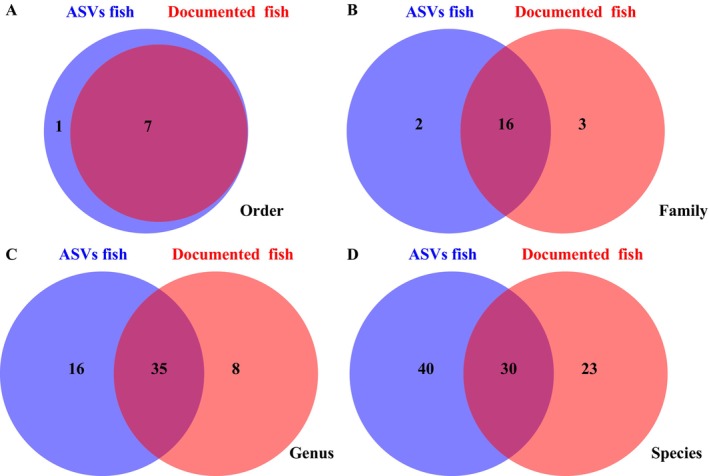
Venn diagrams between ASVs fish and documented fish at Order (A), Family (B), and Genus (C) and Species (D) levels.

In this study, a total of 1,442,955 sequences were analyzed, resulting in the identification of 54,596 ASVs. After removing ASVs with a total read count of less than 5 across all samples and then excluding species that are clearly non‐native (i.e., those endemic to regions outside the study area), 8258 ASVs were retained for subsequent data analysis. From these ASV annotations, 70 species across 51 genera, 18 families, and 8 orders were identified, surpassing the number of documented fish. At the order level, the ASVs fish included an additional order, Acipenseriformes (Figure 1A, Table S1). At the family level, ASVs fish added two families (Acipenseridae and Salmonidae), while shared 16 families with documented fish (84.2%). However, ASVs fish did not include three families: Mastacembelidae, Osmeridae, and Osphronemidae (Figure 1B; Table S1). At the genus level, ASVs fish included 16 additional genera, sharing 35 genera with the documented fish (81.4%), but failed to detect 8 genera: Abbottina, Hypomesus, Macropodus, Onychostoma, Pelteobagrus, Sinobdella, Toxabramis, and Varicorhinus (Figure 1C; Table S1). At the species level, ASV‐identified fish included 40 additional species, shared 30 species with the documented fish (56.6%), but failed to detect 23 species (Figure 1D; Table S1). The shared species accounted for 54% of the relative abundance.

### 3.2 Ichthyological Resources Analysis of Foraging Area Based on ASVs

**FIGURE 2:**
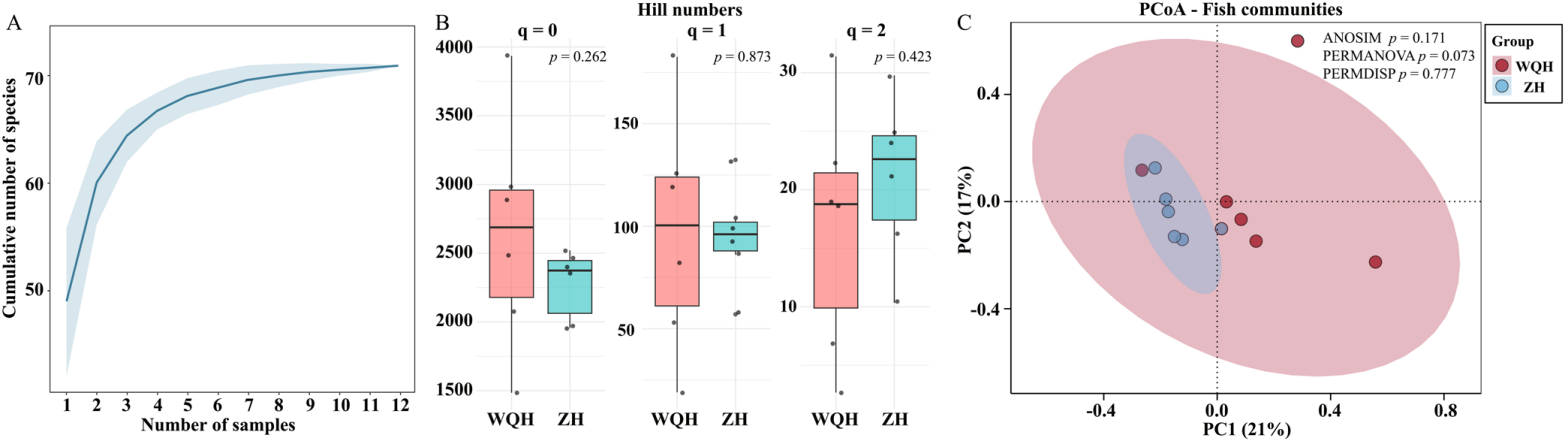
Analysis of fish community diversity. (A) Species accumulation curves showing that sampling coverage was largely sufficient. (B) Hill numbers showed no significant differences between the two groups at *q* = 0, 1, and 2. (C) PCoA analysis revealed no significant difference in fish community composition between the two groups.

Species accumulation curves showed that the cumulative number of detected fish species tended to plateau under the current sampling, indicating that sampling coverage was largely sufficient (Figure 2A). To assess alpha diversity, Hill numbers were calculated for *q* = 0 (species richness), *q* = 1 (Shannon diversity), and *q* = 2 (Simpson diversity). No significant differences were observed between WQH and ZH for any of the three diversity orders (*p* > 0.05), suggesting comparable fish species richness and evenness between the two wetland parks (Figure 2B).

Beta diversity was assessed using PCoA based on Bray‐Curtis distances. The first two axes explained 38% of the total variation in fish community composition, and no distinct separation was observed between WQH and ZH (Figure 2C). Both ANOSIM and PERMANOVA showed that there were no significant differences in community composition between the two groups (*p* > 0.05). Furthermore, PERMDISP revealed no significant difference in within‐group dispersion (*p* > 0.05), confirming that the observed similarity was not simply due to heterogeneous variances. Collectively, these results indicate that fish community composition did not differ significantly between the rivers of the two national wetland parks.

**FIGURE 3:**
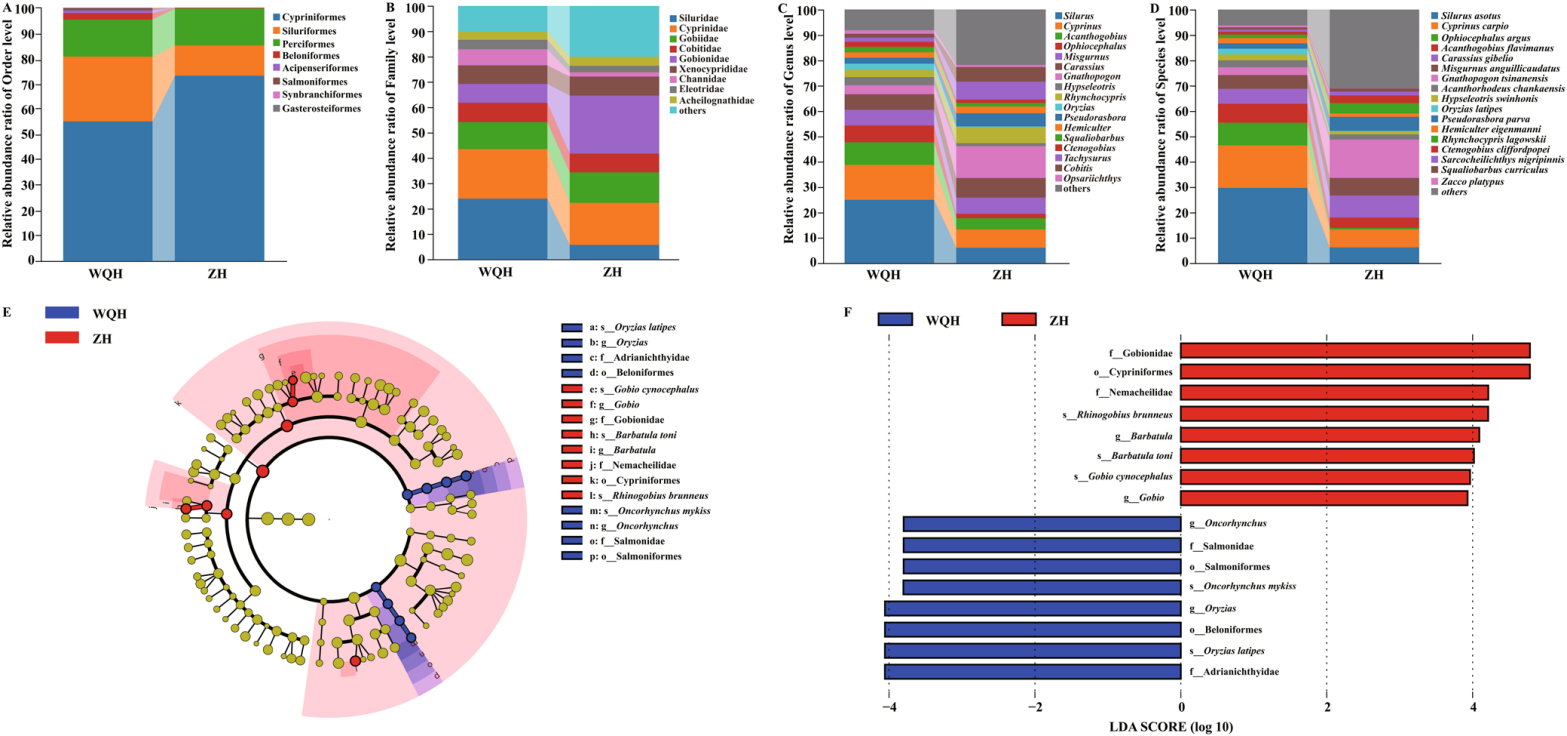
The relative abundance ratios and LEfSe analysis of fish taxa between WQH and ZH. (A–D) Analysis results of the relative abundance ratios of fish taxa at order, family, genus and species levels between WQH and ZH, respectively. (E, F) Cladogram and the LDA score histogram of LEfSe analysis.

Eight orders included the Acipenseriformes, Beloniformes, Cypriniformes, Perciformes, Salmoniformes, Siluriformes, Synbranchiformes, and Gasterosteiformes. Cypriniformes predominated in both regions, and along with Siluriformes and Perciformes, comprised over 95% of the total, representing the principal fish groups. When comparing the composition between the two regions, the proportion of Siluriformes was greater in WQH than in ZH, whereas Cypriniformes were more prevalent in ZH than in WQH (see Figure 3A). At the family level, ASVs identified 18 families in total. Notably, Siluridae from Siluriformes; Cyprinidae, Cobitidae, and Gobionidae from Cypriniformes; and Gobiidae from Perciformes collectively constituted over 60% of the total. Between the two regions, the relative abundance ratio of Siluridae was higher in WQH than in ZH, while Gobionidae was higher in ZH than in WQH (see Figure 3B). At the genus level, ASVs identified a total of 52 genera. The eight most dominant genera, based on relative abundance, were *Silurus*, *Cyprinus*, *Acanthogobius*, *Ophiocephalus*, *Misgurnus*, *Carassius*, *Gnathopogon*, and *Hypseleotris*. When comparing the two regions, the relative abundance ratios of genera such as *Silurus*, *Cyprinus*, *Acanthogobius*, and *Ophiocephalus* were higher in WQH than in ZH, whereas the proportion of *Gnathopogon* exhibited the opposite trend (see Figure 3C). At the species level, ASVs identified a total of 70 species. The relative abundance ratios of *Silurus asotus*, *Cyprinus carpio* , *Ophiocephalus argus*, and *Acanthogobius flavimanus* were higher in WQH than in ZH, whereas *Gnathopogon tsinanensis* exhibited the opposite trend (see Figure 3D). Notably, statistical analysis using *t*‐tests did not reveal significant differences between the two regions at the order, family, genus, and species levels.

Results from the LEfSe analysis (Figure 3E,F) indicated that the WQH region was enriched with 12 significantly differential taxonomic units. The primary species identified included *Oryzias latipes* (order Beloniformes, family Adrianichthyidae) and *Oncorhynchus mykiss* (order Salmoniformes, family Salmonidae). In contrast, the ZH region exhibited enrichment with eight significantly different taxonomic units, predominantly comprising three species: *Rhinogobius brunneus* from the family Gobiidae (order Perciformes), *Barbatula toni* from the family Nemacheilidae, and *Gobio cynocephalus* from the family Gobionidae (both from the order Cypriniformes).

## 4 Discussion

Based on long‐term field observations, Zhanghe Canyon National Wetland Park and Qixi River National Wetland Park in Anyang City, Henan Province, China, are important year‐round habitats for black storks, with a notable increase in their population during the migration period from late October to early November. In the end of October 2024, we recorded 45 individuals in WQH and 18 in ZH. Recognizing that the availability and composition of fish resources directly impact habitat quality and population sustainability, it is imperative to characterize these resources for informed conservation planning. To assess the fish resources available to black storks in these habitats, we employed eDNA metabarcoding technology.

This method revealed a diverse array of fish species, demonstrating the high sensitivity of eDNA metabarcoding for biodiversity assessments. Despite utilizing only a single pair of universal fish primers, the ASVs fish encompassed 100% of the orders, 84.2% of the families, 81.4% of the genera and 56.6% of the species of the documented fish (Zhao et al. 2016; Zhao et al. 2017; Li et al. 2018). However, we also failed to detect the remaining 43.4% of the documented fish. This is primarily due to the limited spatial scope of our sampling. Anyang City encompasses extensive areas within both the Haihe River Basin and the Yellow River Basin, yet our sampling was restricted to only two rivers within the Haihe River Basin, with a limited number of sampling sites located in shallow floodplains. Consequently, species distributed exclusively in the Yellow River Basin or inhabiting deeper downstream habitats, such as reservoirs, were unlikely to be detected. Nonetheless, our sampling was deliberately aligned with the foraging microhabitats of Black Storks, providing a relevant representation of their accessible prey base, rather than a comprehensive inventory of the entire regional fish fauna.

A key aspect of eDNA studies is the management of false‐positive results, which may arise from cross‐amplification of closely related species, non‐specific primer binding, or external contamination by migratory birds (Stoeckle et al. 2016; Nichols et al. 2018). Using highly specific, dedicated primers can mitigate these risks. Despite using primer pairs (MlCOIintF and JgHCO2198) commonly cited in the literature (Kravtsova et al. 2021; Tanaka et al. 2023; Poyntz‐Wright et al. 2024), we still observed some false‐positive signals. In addition, external contamination represents another potential source of non‐native signals. During the peak migration period, piscivorous birds may transport DNA from fish consumed at prior stopover locations via their feces, resulting in the detection of eDNA from non‐local species. To ensure the robustness of our core results, we adopted a two‐step filtering method. Initially, we excluded ASVs with a total read count of less than 5 across all samples to minimize background noise. We then cross‐referenced the retained ASVs with regional fish inventories and their known natural distributions, removing ASVs assigned to species that are clearly non‐native, such as fish from the genus *Schizothorax*, which are indigenous solely to the waters of the Qinghai‐Tibet Plateau in China (Qi et al. 2015). Similarly, we also detected species from the genus *Acipenser* (family Acipenseridae, order Acipenseriformes) likely due to eDNA signals from fish farms or temporary cages established along the riverbanks entering the water system. As these species are not part of the resident fish community in these foraging habitats, they were excluded from the core analysis. This approach mitigated the impact of technical limitations and potential contamination, making the retained ASVs accurately represent the resident fish community in the black stork's foraging habitats.

A comparative analysis between WQH and ZH revealed no significant differences in fish community composition. Alpha diversity, evaluated using Hill numbers (*q* = 0, 1, 2), was similar between WQH and ZH. Furthermore, beta diversity analyses (PCoA, ANOSIM, PERMANOVA, and PERMDISP) corroborated that the community structure did not differ significantly between sites. Given the relatively small sample size (*n* = 12), the absence of statistically significant differences is not unexpected. However, the consistency across multiple analytical approaches supports the conclusion that the fish communities in these two key foraging areas are broadly similar. At both parks, fish species from the orders Siluriformes, Cypriniformes, and Perciformes dominated the relative abundance, including *Silurus asotus*, *Cyprinus carpio* , *Gnathopogon tsinanensis* , *Acanthogobius flavimanus* , and *Ophiocephalus argus*. These taxa represent the core fish available to Black Storks in these parks. LEfSe analysis identified several indicator species, including *Oryzias latipes* and *Oncorhynchus mykiss* in the WQH region, and *Rhinogobius brunneus* , *Barbatula toni* , and *Gobio cynocephalus* in the ZH region. The detection highlights the sensitivity of eDNA in characterizing even less abundant components of fish communities.

In summary, eDNA metabarcoding has proven to be a sensitive tool for characterizing the diversity of potential fish prey within Black Stork foraging habitats. Although this method does not directly quantify fish biomass, it offers a complementary approach to traditional survey techniques, facilitating rapid and non‐invasive assessments of species composition across multiple sites. The present findings demonstrate that the fish communities within Black Storks foraging habitats in Anyang are predominantly by several species from the orders Siluriformes, Cypriniformes, and Perciformes. Given the conservation significance of black stork, it is recommended that local wildlife management agencies integrate dynamic monitoring of these key fish taxa into routine conservation planning for Black Stork habitats. Such monitoring would contribute to ensuring the long‐term stability and sustainability of the essential food resources for this nationally protected species.

## Author Contributions

**Xiangguo Yang:** conceptualization (lead), data curation (lead), investigation (lead), methodology (lead), project administration (lead), writing – original draft (lead), writing – review and editing (lead). **Bo Liu:** formal analysis (lead), investigation (equal), writing – original draft (supporting). **Jiangyue Wu:** formal analysis (equal), investigation (equal), validation (equal), visualization (lead). **Xueyong Yuan:** investigation (equal), visualization (equal). **Danyang Li:** investigation (equal), visualization (equal). **Sen Liu:** conceptualization (lead), data curation (equal), investigation (lead), supervision (lead), writing – review and editing (lead).

## Funding

This work was supported by the Black Stork Resource Survey Project within the Jurisdiction of Anyang City in the National Key Protected Wild Animals and Plants Project under the 2022 China Central Finance Forestry and Grassland Project Reserve Database, and the Research Fund for Doctoral Talents of Henan Normal University (QD2021003).

## Conflicts of Interest

The authors declare no conflicts of interest.

## Supporting information


**Table S1:** Species in ASVs fish and documented fish.

## Data Availability

The sequence associated with this research has been deposited into the Genome Sequence Archive (GSA: CRA034477) in the National Bioinformation Center of China.
